# Prognostic factors, survival outcomes, and surgical practices when dealing with uterine sarcomas: 8 years’ clinical experience

**DOI:** 10.4274/jtgga.galenos.2019.2019.0061

**Published:** 2019-08-28

**Authors:** Elif Meseci, Mehmet Murat Naki

**Affiliations:** 1 Clinic of Obstetrics and Gynecology, Acıbadem Kozyatağı Hospital, İstanbul, Turkey; 2 Department of Obstetrics and Gynecology, Acıbadem University School of Medicine, İstanbul, Turkey

**Keywords:** Carcinosarcoma, leiomyosarcoma, prognosis, sarcoma, survival

## Abstract

**Objective:**

To determine the clinical and pathologic characteristics, prognostic factors, surgical practice, adjuvant therapies, and survival outcomes of patients with uterine sarcoma diagnosed and treated in our institution.

**Material and Methods:**

Patients diagnosed and treated for uterine sarcomas at our institution from 2009 to 2017 were retrospectively evaluated. All histologic slides from the specimens underwent a thorough pathologic review by a gynecologic pathologist. The following variables were assessed: age, family history of cancer, smoking status, age of menarche, parity, age at first delivery, related symptoms, clinical staging, histologic type, treatment received, disease-free period, and the time and site of recurrence, as well as treatment of the latter and overall survival.

**Results:**

Ten patients were diagnosed as having leiomyosarcoma, a further 10 patients had malignant mixed mullerian tumors, and five had endometrial stromal sarcoma; the remaining nine patients had other tumors. At the end of our study, 12 (35.3%) patients were alive and in remission, four (11.8%) were alive with disease, 10 (29.4%) were lost to follow-up, and eight (23.5%) had died. The mean survival time was 80.92 months, and the 2-year survival rate was 75.6%. We found that survival was significantly shorter in the presence of lymph node involvement, residual tumor, and recurrence.

**Conclusion:**

This study serves to inform physicians about the outcome of various uterine sarcomas that were diagnosed and managed at our center. We found that 35.3% of our patients were alive and in remission, 11.8% were alive with disease, 29.4% were lost to follow-up, and 23.5% of patients died.

## Introduction

Uterine sarcomas are malignant tumors that originate from the mesodermal tissues (muscle and supportive tissues) of the uterus. They are usually of heterogeneous characteristics and represent a small group among the malignant neoplasms of the uterus (1,2). The prevalence of uterine sarcoma is between 1.5 and 3 cases per 100,000 for Caucasians and Afro-Americans, respectively (3).

The World Health Organization (WHO) classifies uterine sarcomas into two types: (1) malignant mesenchymal tumors, and (2) mixed epithelial and mesenchymal tumors. Pure mesenchymal tumors are further subclassified as leiomyosarcoma (LMS), low- and high-grade endometrial stromal sarcomas (LG-ESS and HG-ESS, respectively) and undifferentiated uterine sarcoma (UUS) (4). Among these, LMS is the most frequently seen type with a frequency of 60-70% among all uterine sarcomas; the remaining 3 subtypes (LG-ESS, HG-ESS and UUS) collectively comprise another 10% of uterine sarcomas (5). Mixed tumors comprise adenosarcoma (AS), rhabdomyosarcoma (RMS), and perivascular epithelioid cell neoplasms (PEComa) (4,6). These are much rarer, collectively representing around 5% of all uterine sarcomas (7).

Although dependent on tumor type, uterine sarcomas are most commonly seen between the 5^th^ and 7^th^ decade of life. Risk factors for uterine sarcoma development have been identified as obesity, diabetes, having undergone previous pelvic irradiation therapy and/or tamoxifen treatment, and having excessively high or unopposed estrogen levels (8,9,10,11,12). However, data are scarce on this topic due to the rarity of uterine sarcomas; therefore, there is no universal consensus on risk factors, optimal therapeutic approaches, and the frequency of poor outcomes. Our aim in this study was to determine the clinical/pathologic characteristics, prognostic factors, surgical practices, adjuvant therapies, and survival outcomes of patients who received treatment for uterine sarcoma at our institution.

## Material and Methods

Our study was a retrospective evaluation of the medical files of patients who were diagnosed as having uterine sarcomas and treated at our institution from 2009 to 2017. The study was approved by the local Ethics Committee (reference number: 2017-16/28). All histologic slides underwent a thorough pathologic review by a gynecologic pathologist. Staging was performed according to the current International Federation of Gynecology and Obstetrics (FIGO) criteria (13).

Our patient group also included those who had been diagnosed as having uterine carcinosarcomas [also called malignant mixed mullerian tumors (MMMT)] because these tumors are now classified within the uterine carcinoma group, having previously been considered as uterine sarcomas (14,15). Also of note, four patients in which endometrial sampling had not detected malignancy, but hysterectomy results were conclusive of uterine carcinoma (1 LMS, 1 MMMT, 1 LG-ESS, 1 AS), were also included in the study. Two patients who were initially diagnosed as having LG-ESS, but were found to have endometrial stromal nodule and high-grade serous carcinoma after hysterectomy, were excluded from the study. Patients with metastatic sarcoma from other gynecologic sites and those who had incomplete data for demographic analyses were excluded from the study.

All remaining patients who were confirmed to have uterine sarcomas were included in the study; however, those without sufficient data in terms of clinical findings, pathologic results, follow-up studies, and treatment approach/results were excluded from the survival analysis. The following characteristics of all patients were assessed and recorded: age, parity, age at first delivery, age at menarche, family history of cancer, smoking status, and other related symptoms. In regard to disease characteristics, the following were assessed from medical records: clinical stage, histologic type, treatment approach, disease-free period, overall survival (OS), and the time and site of recurrence.

Patients were grouped according to the following parameters: tumor size (≤5 cm, >5 cm), FIGO stage [early (I-II), advanced (III-IV)], histologic grade (low, moderate, high), myometrial invasion (absent, <50%, ≥50%). In addition, Ki-67 positivity was also evaluated on a present/absent basis with a cut-off of 14%.

The treatment plan of each patient was structured according to the most recent protocols and guidelines with regard to tumor stage/grade, age, and cell type. The use of adjuvant therapies such as chemotherapy, radiotherapy or immunotherapy were also based on the most recent guidelines. All surgical interventions were performed by our Gynecology Department and lymphadenectomies were performed according to the discretion of the primary surgeon in each operation.

Disease free survival (DFS) was defined as the period of time (in months) from diagnosis to either recurrence or last follow-up. OS was defined as the period of time (also in months) between diagnosis to either the date of death or last follow-up.

### Statistical analysis

All statistical analyses were performed using the SPSS version 21 software for the Windows operating system (IBM, Armonk, NY, USA). Continuous variables are given as mean ± standard deviation, and categorical variables are presented with frequency (n) and percentage (%). The DFS and OS analyses were performed using the Kaplan-Meier method. The comparison of survival times between groups was performed using the log-rank test. Cox-regression analysis with the Backward conditional method was used to determine the effects of continuous and categorical variables on survival times. P values less than 0.05 were accepted to show statistical significance.

## Results

The mean age of the 34 patients included in our study was 52.56±14.47 years. Ten patients had LMS, 10 patients had MMMT, five patients had ESS, and nine patients had other types of tumors (5 with AS, 3 with UUS, 1 with embryonal rhabdomyosarcoma). Patients with MMMT were found to have a higher mean age compared with the other groups (62.40±7.97 years vs 49.80±5.87 years in LMS, 39.60±13.22 years in ESS, and 51.89±20.74 years in other sarcomas). Age difference was only significant when the MMMT and ESS groups were compared (p=0.016). The mean follow-up duration of the patients was 31.1±31.1 months.

FIGO staging revealed that 22 patients (64.7%) were stage I, seven patients (20.6%) were stage II, one patient (2.9%) was stage III, and four patients (11.8%) were stage IV. The majority of our patients (67.6%) were post-menopausal and had presented with bleeding (73.5%). The median primary tumor size was 6 cm (minimum-maximum: 2-15 cm). There were no significant differences between the groups in regard to tumor size (p=0.845). Nineteen patients had undergone pelvic and/or paraaortic lymph node dissection and only one patient (in the MMMT group) was found to have a positive lymph node. Nineteen (55.9%) patients received at least one kind of adjuvant therapy; six received adjuvant chemotherapy, five received radiotherapy, two received hormono therapy, and six received chemotherapy and radiotherapy in sequence. The most common chemotherapy drugs used were carboplatin + paclitaxel. Three patients were found to have residual tumor after surgery, and 14 patients had recurrence. The pelvic peritoneum was the most common site of recurrence in these patients. At the final follow-up, 12 (35.3%) patients were alive and in remission, four (11.8%) were alive with disease, 10 (29.4%) had been lost to follow-up, and 8 (23.5%) had died (Table 1).

**Figure 1:**
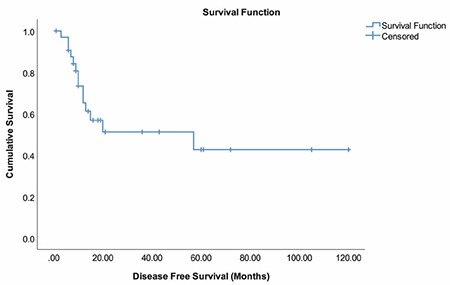
Disease-free survival times of patients

**Figure 2:**
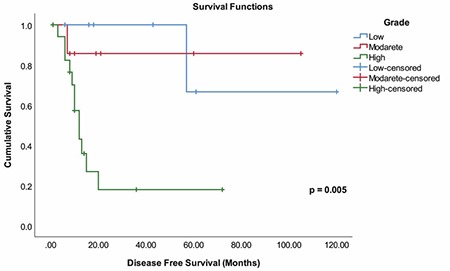
Disease-free survival times by tumor grade

The mean DFS was 61.21±11.11 months (Figure 1). DFS was significantly higher for patients with early FIGO stages (p=0.030). Tumors with high histologic grade had shorter DFS times compared with the low and moderate grades (p=0.005) (Figure 2). We found that DFS was significantly decreased in patients with lymphovascular involvement (p=0.015) and those with positive lymph nodes (p<0.001). We also found that those with residual tumor and positive Ki-67 indexes had shorter DFS; however, these results were not found to be significant. Receiving adjuvant therapy was found to have no significant effect on DFS (p=0.490) (Table 2).

**Figure 3:**
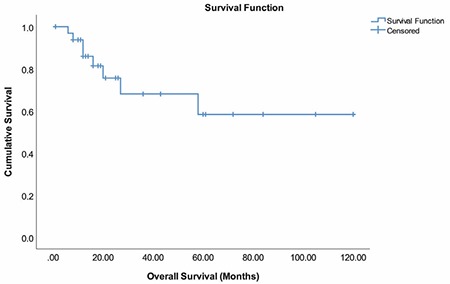
Overall survival times of patients

**Figure 4:**
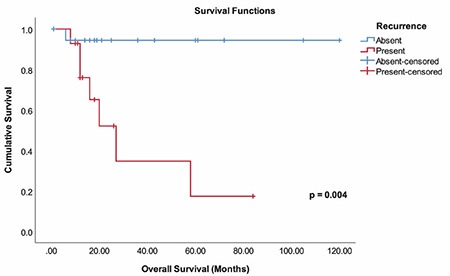
Overall survival times by recurrence

The mean survival time was 80.92±11.46 months and the 2 year survival rate was 75.6% (Figure 3). Survival times were significantly shorter in patients who were found to have positive lymph nodes (p=0.048), those with residual tumor (p<0.001), and those with recurrence (p=0.004) (Figure 4). We also found that patients with at least one parity, early (FIGO I and II) stages, and low histologic grade had longer survival times overall, but these results were not statistically significant (Table 3).

After performing the Cox regression analysis, we found that age and parity had no significant effect on DFS times. However, those who were older at menarche had a 2.2-times higher risk for recurrence and those who were older at first delivery were found to have a 1.9-fold greater risk for recurrence. Additionally, larger tumor size also incurred a 1.5-fold higher (for each cm) risk for recurrence (Table 4). We found no significant effect on survival rates when we took into account age, age at menarche, and age at first delivery (Table 5). Furthermore, we found that larger tumor sizes decreased survival rates but this result was deemed statistically insignificant.

## Discussion

Uterine sarcoma is rare and difficult to study; therefore, it features very little in the current medical literature. This study was made up of 34 patients who were referred over an 8 year period. Histopathologic evaluations revealed that LMS and MMMT occurred in equal frequency in our group of patients (29.4%), followed by ESS (14.7%). Our data are comparable to some studies (7,16), but at the same time there are studies reporting very different histopathologic distributions in their results (17,18,19). It should be noted that small numbers of patients and changes in the WHO classification in each study may have caused these differences.

The mean age of our patients were 62.4 years in those with MMMT, 49.8 years in those with LMS, 39.6 years in those with ESS, and 51.8 years in other sarcomas types. Our findings are consistent with the study by Benito et al. (17) and Potikul et al. (18), with the only exception being the ESS group, which was younger in our study.

In the current study, only 7 cases of uterine sarcoma were diagnosed in patients aged under 40 years and the majority of cases were seen in postmenopausal women. Although RMS is usually associated with the pediatric age group (20), one patient was diagnosed at the age of 31 years. Another patient’s diagnosis was made during cesarean section by ovarian biopsy, which revealed a high-grade UUS. At the time of diagnosis, metastases had already developed in the lung, brain, and liver.

One patient had a personal history of breast cancer, and four had concomitant malignancies associated with MMMT; one gastrointestinal stromal tumor, two low-grade uterine endometrioid adenocarcinomas, and one high-grade ovarian serous adenocarcinoma. Family history for cancer was positive for a total of 6 (17%) patients, with breast carcinoma being the most commonly reported type. None of the patients had a personal or family history of sarcoma, nor did they report any history of pelvic irradiation. One patient (2.9%) who had a prior history of breast carcinoma had received treatment with tamoxifen. Durnali et al. (21) reported tamoxifen treatment frequency as 1% in their study. Benito et al. (17) reported a higher incidence of a positive family history (40.4%), and prior histories of cancer were similar to those reported by Benito et al. (17) and Koivisto-Korander et al. (22) in their studies (10.1% and 11%, respectively), with breast carcinoma as the most common. Similar to our study, these studies also reported that none of their patients had a history of pelvic irradiation. Wais et al. (19) and Durnali et al. (21) reported a lower occurrence of personal cancer history among their patients (8% and 3%, respectively), and a history of pelvic irradiation was reported in only 1%.

A correct preoperative malignancy diagnosis was achieved in 17 of our patients (73.9%). Some studies have reported higher (86-88%) rates of preoperative diagnosis, whereas others reported lower rates (65%, 64%) (18,19,23). Bansal et al. (23) correctly predicted the presence of invasive tumors in 86%, while also correctly predicting the histologic subtype in 64% of their patients. Some differences in preoperative diagnostic methods may have resulted in variable results.

In this patient group, complete resection of the uterus and removal of both adnexa is the widely accepted approach to treatment of early-stage disease. It is suggested to avoid pelvic and para-aortic lymphadenectomy when unremarkable, except in patients with MMMT (5). In cases of MMMT limited to the uterus, positive lymph nodes are reported in around 30% of patients. The literature on this topic reports that OS is adversely effected by systematic lymph node involvement (5). In our study, we found that the mean number of lymph nodes that were removed was 18.9±22.4; this value was 15.1±17.4 for pelvic lymph nodes and 12.6±9.2 for paraaortic lymph nodes. According to pathology reports, one of the pelvic lymph nodes demonstrated high-grade MMMT (FIGO 3C). The OS time of this patient was 12 months. However, the literature on this topic reports higher lymph node metastasis rates. In the current study, lymph node metastases were not found in any patients with other types of sarcoma. The number of patients with positive lymph nodes was low in our study, and survival times were found to be significantly shorter for those with positive lymph nodes.

In patients with LMS limited to the uterus, the ovaries of women of childbearing age may be preserved (24,25). Additionally, preservation of the ovaries was not found to impact OS negatively in patients with LG-ESS; however, it is crucial to consider removal of ovaries on a case-by-case basis because LG-ESS is known to be an endocrine-driven tumor (26). The preservation of ovaries was performed in only five patients in the current study. One of these patients had AS and underwent TAH + BPLND, but was later (6 months) found to have adnexal metastasis. The lesion was subsequently excised and palliative chemotherapy was recommended. Eighteen months after the initial treatment she was lost to follow-up because she had settled overseas. Another patient had botryoidal-type embryonal rhabdomyosarcoma at the time of diagnosis and was pregnant. She gave birth through cesarean section at 35 weeks of gestation after confirmation of fetal lung maturation, and later underwent radical hysterectomy + BPPALND + oophoropexy with postoperative adjuvant chemotherapy (vincristine and actinomycin D). She is still alive without any evidence of disease at 105 months of follow-up. Two patients who had undergone hormone therapy were still alive at 16 and 121 months of follow-up. Brain metastasis occurred at the seventh month in a patient with LMS whilst receiving chemotherapy with the survival time being months. Due to the limited number of patients, it is difficult to make any recommendation for ovarian preservation.

In our study, most patients were diagnosed at an early stage (85.3% were diagnosed at FIGO stages I and II). This rate is higher compared with other studies, which reported rates between 58 and 66% for early-stage disease diagnosis (17,18,21,22). In contrast to our results, MMMT was most often diagnosed during advanced stages (17,18,21). However, in our study, only 20% of MMMT cases were detected at an advanced stage. These differences may be explained by the extent of the operative procedure, the extent and type of sarcoma, and the newer FIGO staging system that we used. Given these differences, it may not be feasible to compare our study with prior studies on this field.

In patients with uterine sarcomas, the role of adjuvant therapy on survival is uncertain (7). Studies show that adjuvant chemotherapy has a positive effect on survival in MMMT and LMS (increasing OS and DFS), and receiving pelvic irradiation was associated with significantly longer OS in those with ESS and UUS (27,28). In a large study comprising 3650 patients with uterine sarcoma (MMMT, ESS, LMS and UUS), it was shown that adjuvant pelvic radiotherapy reduced local-regional failure in up to 53% of cases (29). Durnali et al. (21) showed that adjuvant radiotherapy after chemotherapy for uterine sarcomas improved DFS but had no effect on OS. In our present study, adjuvant therapy did not seem to improve OS. However, due to the low number of patients in our study, it would be unfeasible to draw conclusions in regard to the efficacy of adjuvant treatments.

Uterine sarcomas have a poor prognosis overall. Our results show the recurrence rate as 41.1% for patients with uterine sarcoma with a median follow-up time of 61.2 months. Previous reports of recurrence rates have been reported to range between 36% and 63.4% (16,17,18,21,30). In the current study, the following factors were found to contribute to significantly poor prognosis: later FIGO staging, higher tumor grade, lymphovascular space invasion, and lymph node involvement. We also found that the presence of residual tumor and positive Ki-67 decreased DFS; however, the decreases were not statistically significant for either comparison, presumably due to the low number of patients. However, our findings were in agreement with a few previous studies (18,30). It should also be mentioned that higher age at menarche and higher age at first birth were associated with recurrence, which are strongly considered as being risk factors for UUS (31).

The mean OS in our study was found as 80.92 months, and the 2-year survival rate was 75.6%. In previous studies, the 2-year OS has been reported within a range of 49-69%, and 5-year OS is reported as 45-59% (16,17,21,30). According to our results, survival times were significantly shorter in those with lymph node involvement, residual tumor, and tumor recurrence. We also found that patients with at least one parity, early FIGO (I & II) stages, and low histologic grade had longer survival.

There are limitations to our study. First, it is evident that our findings should be interpreted in the context of the limitations associated with retrospective studies. Secondly, the number of cases was low; however, uterine sarcomas are rare and the fact that the study was cconducted in a single center with rigorous inclusion/exclusion criteria further limited the number of patients that could be included in the study. Lastly, the number of patients lost to follow-up due to various reasons can be considered as another limitation of the study. In regard to these limitations, our results concerning the survival of these patients must be evaluated with caution.

In conclusion, at the final follow-up of the current study, 35.3% of patients were alive and in remission, 11.8% were alive with disease, 29.4% were lost to follow-up, and 23.5% had died. The mean survival time was 80.92 months and the 2-year survival rate was found as 75.6%. According to our results, survival times were significantly shorter with lymph node involvement, the presence of residual tumor, and tumor recurrence. We also found that patients with at least one parity, early FIGO stages (I & II) and low histologic grade had longer survival times. Considering the low incidence of uterine sarcomas and because of the recent changes in the classification system, it is very difficult to reach conclusions in terms of treatment strategies.

## Figures and Tables

**Table 1:**
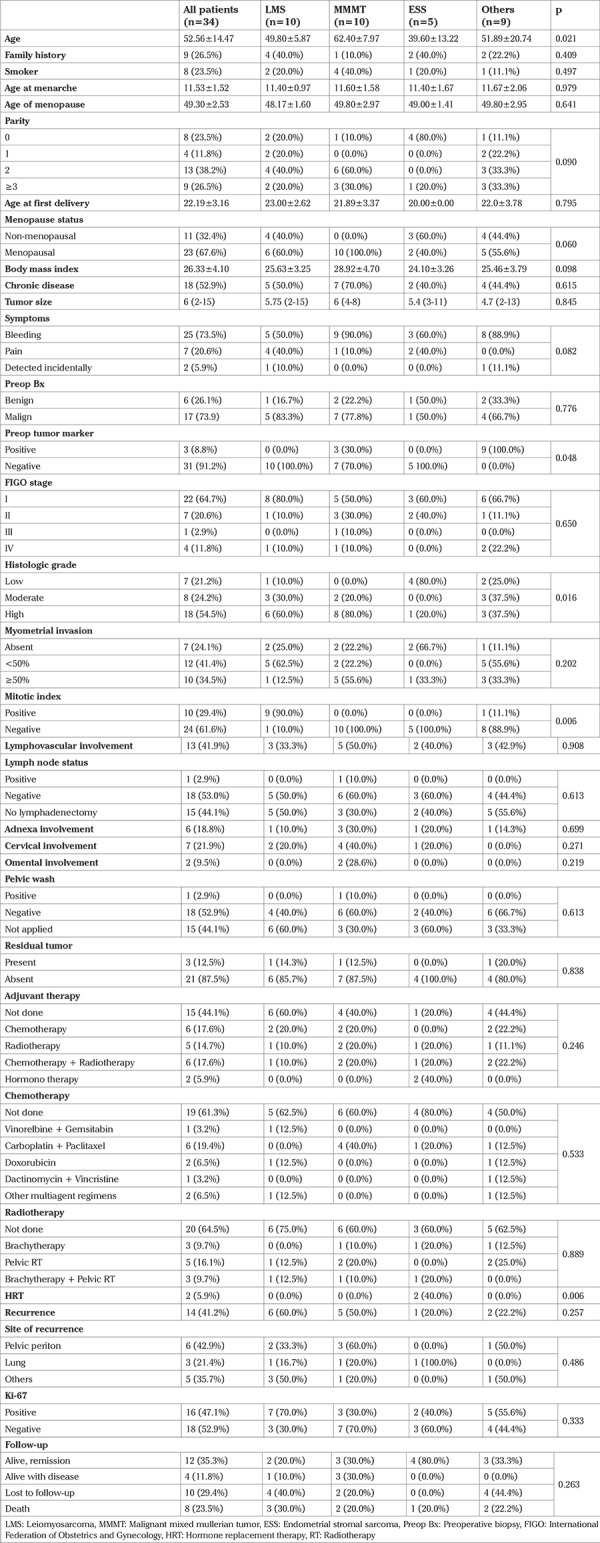
Summary of our variables

**Table 2:**
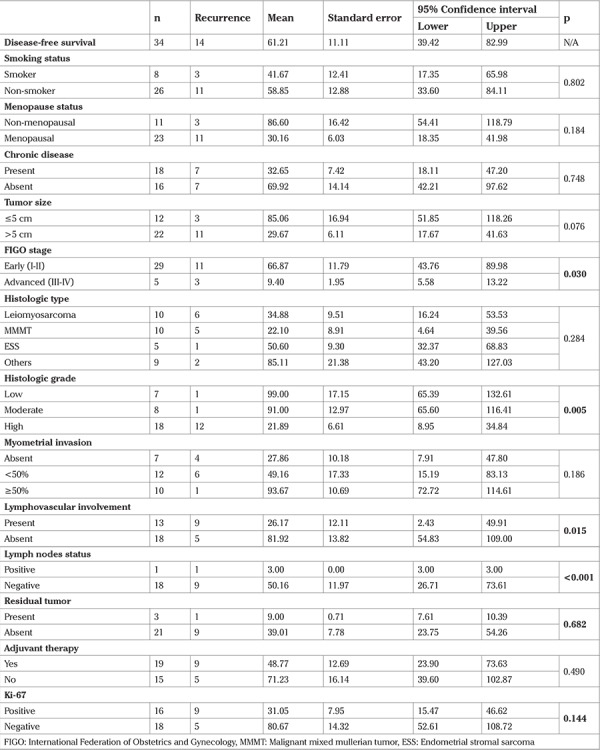
Disease-free survival times (months) with Kaplan Meier method and comparisons of groups using the Log-rank test for categorical variables

**Table 3:**
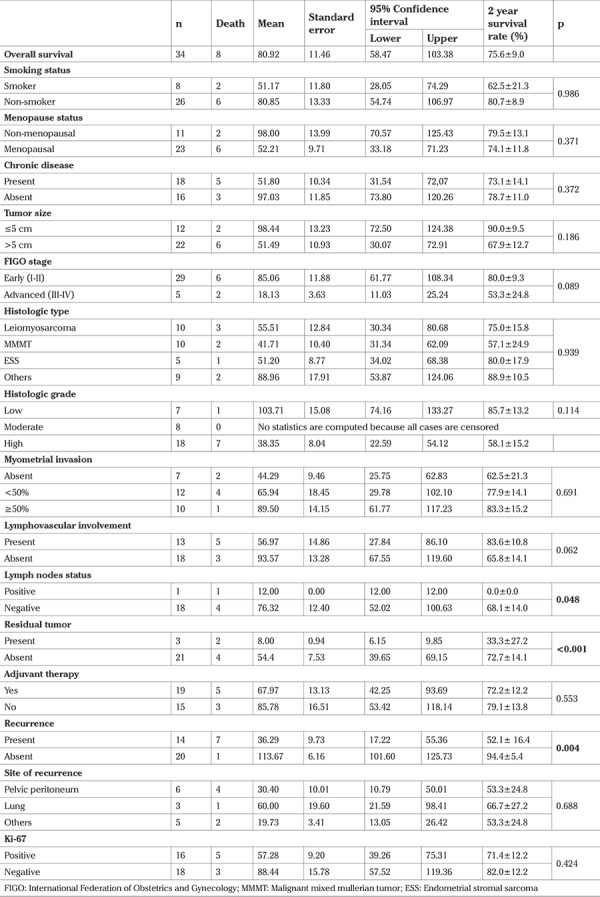
Survival times (months) using Kaplan-Meier analysis and comparisons of groups using the Log-rank test for categorical variables

**Table 4:**
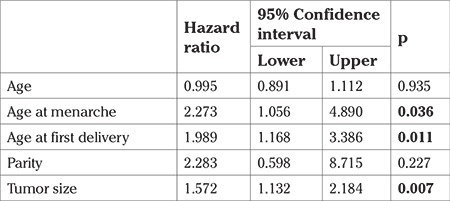
Cox regression analysis results for disease-free survival times (months)

**Table 5:**
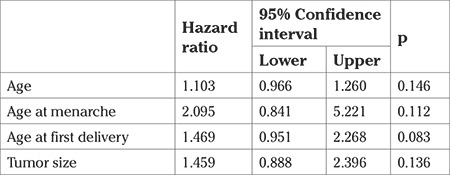
Cox regression analysis results for survival times (months)
